# Exploring equity in uptake of the NHS Health Check and a nested physical activity intervention trial

**DOI:** 10.1093/pubmed/fdv070

**Published:** 2016-10-17

**Authors:** S. Attwood, K. Morton, S. Sutton

**Affiliations:** 1 UKCRC Centre for Diet and Activity Research (CEDAR), MRC Epidemiology Unit, University of Cambridge School of Clinical Medicine, Cambridge CB2 0QQ, UK; 2 Behavioural Science Group, Primary Care Unit, Institute of Public Health, University of Cambridge, Cambridge CB2 0SR, UK

**Keywords:** physical activity, public health, socioeconomic factors

## Abstract

**Background:**

Socio-demographic factors characterizing disadvantage may influence uptake of preventative health interventions such as the NHS Health Check and research trials informing their content.

**Methods:**

A cross-sectional study examining socio-demographic characteristics of participants and non-participants to the NHS Health Check and a nested trial of very brief physical activity interventions within this context. Age, gender, Index of Multiple Deprivation (IMD) and ethnicity were extracted from patient records of four General Practices (GP) in England.

**Results:**

In multivariate analyses controlling for GP surgery, the odds of participation in the Health Check were higher for older patients (OR 1.05, 95% CI 1.04–1.07) and lower from areas of greater deprivation (IMD Quintiles 4 versus 1, OR 0.37, 95% CI 0.18–0.76, 5 versus 1 OR 0.42, 95% CI 0.20–0.88). Older patients were more likely to participate in the physical activity trial (OR 1.04, 95% CI 1.02–1.06).

**Conclusions:**

Younger patients and those living in areas of greater deprivation may be at risk of non-participation in the NHS Health Check, while younger age also predicted non-participation in a nested research trial. The role that GP-surgery-specific factors play in influencing participation across different socio-demographic groups requires further exploration.

## Introduction

Vascular diseases, including coronary heart disease, stroke, diabetes and kidney disease, affect more than four million people in England and are responsible for one in every three deaths and one in five hospital admissions annually.^1^ Physical inactivity is a key modifiable risk factor for vascular diseases^2^ and is often targeted in primary care-based interventions delivered to diverse patient groups.^3–8^

An example of one such intervention is England's National Health Service (NHS) Health Checks programme.^9^ This programme invites eligible patients aged 40–74 years to attend a vascular disease risk assessment, usually based in primary care. This assessment incorporates brief advice encouraging physical activity and dietary change in patients considered to be at increased risk. How best to promote physical activity within this time and resource-limited context is the focus of an ongoing randomized controlled trial^10^. The present study uses data from the pilot phase of this trial.

The desired outcome of the Health Check—a reduction in the incidence of vascular diseases in England^11^—will only occur if the programme can successfully recruit and screen large numbers of currently healthy patients. It is widely recognized that population-level preventative interventions like vascular screening may impact minimally on individual recipients, many of whom will be of low risk, yet remain effective at the population level given their potential to reach large numbers (the ‘prevention paradox’).^12^ As Geoffrey Rose explains in his seminal paper ‘Sick Individuals and Sick Populations’, small reductions in a risk factor that occur en masse will prevent more cases of a disease than sizeable changes occurring within high-risk subgroups.^12^

However, Rose's approach to disease prevention has been critiqued by those who suggest that population-level interventions may inadvertently increase inequities in health behaviour and outcomes.^13^ If selection bias operates such that low-risk individuals primarily attend Health Checks and use the support on offer, there is potential for inequities in health to be widened rather than narrowed as a result of the programme.^14,15^ Health inequity has previously been defined as systematic disparities in health between population groups with different levels of underlying social disadvantage or advantage (for example, as a result of their gender, ethnicity, age or socioeconomic status; SES).^16^

To date, a number of socio-demographic factors have been shown to influence uptake of primary care-based preventative interventions. For example, one previous study found that non-participants to a coronary heart disease screening programme were older and of lower SES than participants.^17^ These non-participants were also in poorer health and engaged in fewer health-promoting behaviours than participants,^17^ a finding that implies inequities in health may increase following screening roll out. Other studies of non-participants have observed similar results for SES across a range of different screening interventions,^18–20^ summarized succinctly in two reviews available on this topic.^21,22^ In the context of the NHS Health Check programme specifically, age, ethnicity, gender and SES have all previously been shown to influence non-participation, although the effect of each characteristic may differ with setting.^23,24^ For physical activity promotion interventions, socio-demographic differences in participation have been found in directions that suggest post-intervention increases in health inequities,^25,26^ although contradictory findings are present.^27,28^ For example, considering gender, men have been shown to be both more and less likely than women to participate in physical activity promotion interventions.^25,26,28^

For the present study, we aimed to explore the socio-demographic characteristics of participants and non-participants to the Health Check and a nested research trial of very brief interventions for physical activity. We examined age, gender, ethnicity and deprivation level to draw some initial conclusions regarding equity in uptake.

## Methods

### Design

A cross-sectional design using recruitment data collected as part of a randomized controlled pilot trial. This trial formed one phase of a research programme determining the potential efficacy, fidelity, feasibility and acceptability of very brief interventions to promote physical activity within the Health Check consultation.^29^ Details of the trial are available online.^10^

### Setting

Four General Practice (GP) surgeries in the East of England.

### Participants

Eligible patients were those invited to attend a Health Check and participate in a nested physical activity trial. Recruitment methods differed across surgeries (see Table 1), but involved one or more of the following: mailed invitation letters, mailed reminder letters, face-to-face recruitment of eligible patients attending pre-existing GP appointments and telephone recruitment.

**Table 1:** Details of participating GP surgeries

*GP surgery*	*Registered persons*	*Location*	*Practice IMD Score (National Decile^a^)*	*Practice % non-white ethnicity estimate*	*Recruitment procedures*	*Total number of patients invited to participate (*n* = 1380)*
Practice 1	7000–7999	Urban Town	19.8 (5th decile)	0.0%	Mailed invitations Mailed reminders Face-to-face (in-practice) Telephone	687
Practice 2	9000–9999	Urban Town	18.7 (5th decile)	-	Mailed invitations Face-to-face (in-practice)	215
Practice 3	2000–2999	Urban Town	7.8 (1st decile)	1.8%	Mailed invitations Mailed reminders Face-to-face (in-practice)	380
Practice 4	4000–4999	Rural Town	4.9 (1st decile)	1.7%	Mailed invitations	98

Data on Practice IMD and non-white ethnicity estimate derived from Public Health England.^33^

^a^Decile of deprivation, UK ranking (2012). 1 = least deprived, 10 = most deprived.

### Ethical approval

Ethical approval was obtained from London Harrow Research Ethics Committee (13/LO/1163), with additional approval to access anonymized information on non-participants from GP surgery records obtained from the Health Research Authority Confidentiality Advisory Group (CAG 7-06(d)/2013).

### Measures

Data routinely collected by GP surgeries were compiled for analysis by a member of the practice staff, including patient age (years), sex, ethnicity (16-category UK Office for National Statistics tool)^30^ and area-level SES (Index of Multiple Deprivation; IMD).^31^ The IMD ranks geographical units known as Lower Layer Super Output Areas (areas containing between 400 and 1200 houses, determined by postal code)^32^ based on various domains including income, employment, health, education, crime, access to services and the living environment. IMD scores were categorized into quintiles for analysis, with Quintile 1 corresponding to areas of lowest deprivation.

**Fig. 1:**
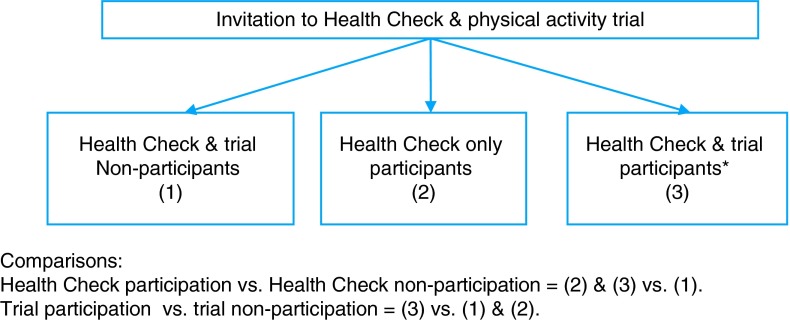
Participant and non-participant comparisons.

The main outcome variables of interest were participation in the Health Check and participation in the nested physical activity trial. Health Check participation included patients who took part in the Health Check only and those who participated in both the Health Check and the trial. Trial participation included patients who took part in both the Health Check and physical activity trial (It was not possible to participate in the trial without also taking part in a Health Check.). Figure 1 clarifies participant and non-participant classifications.

### Analysis

Univariate binary logistic regression analyses were conducted to compare age, gender, ethnicity and IMD scores of Health Check participants versus Health Check non-participants and of trial participants versus trial non-participants (see Fig. 1). Following this, interactions were explored and multivariate binary logistic regression analyses conducted including all socio-demographic variables as predictors and adjusting for GP surgery as a probable confounding factor (There were differences in socio-demographic composition and in recruitment procedures across practices.).

## Results

Four GP surgeries provided data on Health Check and trial participants and non-participants. Table 1 provides further details of participating practices.^33^ These served between 2000 and 10 000 patients in relatively racially homogeneous areas of moderate to low levels of deprivation. All participating GP surgeries used more than one recruitment strategy, with mailed invitation letters the most commonly employed.

In total, 373 patients (27% of *n* = 1380) were classified as Health Check participants (data were missing on Health Check only participants in Practice 2), and 194 (14.1%) patients were classified as physical activity trial participants. Participation rates varied significantly across practices, with Health Check uptake of 37.0, 14.5 and 12.2% seen in Practices 1, 3 and 4 (*χ*^2^ = 74.61, df = 2, *P* < 0.005), and trial uptake rates of 11.4, 24.2, 13.7 and 12.2% seen in Practices 1, 2, 3 and 4 (*χ*^2^ = 22.72, df = 3, *P* < 0.005), respectively. Table 2 presents details of the demographic characteristics of participants and non-participants.

**Table 2:** Demographic details of participants and non-participants

*Variable*	*Unit*	*Invited patients (*n* = 1380)*	*Health Check and trial non-participants (1) (*n* = 844)*	*Health Check only participants (2) (*n* = 179)*	*Health Check and trial participants (3) (*n* = 194)*
Age					
Years	Mean (SD)	52.4 (9.3)	52.0 (9.0)	56.6 (9.9)	54.4 (9.3)
Gender					
Male	*n* (%)	686 (49.7)	427 (50.6)	76 (42.5)	80 (41.2)
Female		694 (50.3)	417 (49.4)	103 (57.5)	114 (58.8)
Ethnicity^a^					
White	*n* (%)	1006 (72.9)	585 (69.3)	144 (80.4)	163 (84.0)
Other		45 (3.3)	19 (2.3)	3 (1.7)	10 (5.2)
IMD^b^ Score	Median (Range)	14.2 (13.8)	13.3 (13.6)	18.3 (9.3)	13.8 (13.9)
Quintile 1 (least deprived)	*n* (%)	275 (19.9)	189 (22.4)	1 (0.6)	48 (24.7)
Quintile 2		319 (23.1)	216 (25.6)	37 (20.7)	43 (22.2)
Quintile 3		235 (17.0)	130 (15.4)	40 (22.3)	35 (18.0)
Quintile 4		285 (20.7)	179 (21.2)	52 (29.1)	32 (16.5)
Quintile 5 (most deprived)		256 (18.6)	125 (14.8)	48 (26.8)	34 (17.5)

^a^White Ethnicity (White British, White Irish, Other White Background); Other Ethnicity includes Mixed Ethnicity [Mixed White and Black Caribbean, Mixed White and Asian, Other Mixed Background, Asian Ethnicity (Asian or Asian British Indian, Asian or Asian British Pakistani, Asian or Asian British Bangladeshi, Other Asian Background), Black Ethnicity (Black or Black British Caribbean, Black or Black British African, Other Black Background), Chinese and Other].

^b^Index of Multiple Deprivation cut-off values for Quintile 1 = 6.12, Quintile 2 = 12.51, Quintile 3 = 16.23, Quintile 4 = 22.41.

### Health Check participants versus Health Check non-participants

Table 3 displays results of univariate and multivariate logistic regression analyses predicting Health Check participation (*n* = 1165). For univariate analyses, with every year of increased age, patients showed a statistically significant 5% increase in the odds of Health Check participation (OR 1.05, 95% CI 1.04–1.07), while the odds of participation were around 50% higher for women than men (OR 1.50, 95% CI 1.16–1.95). Uptake did not differ by patient ethnicity (OR 0.59, 95% CI 0.21–1.57). Using the lowest IMD quintile (least deprived) as the reference group, the odds of taking part in the Health Check were higher in patients residing in more deprived Quintile 2 (OR 1.61, 95% CI 1.03–2.51), Quintile 3 (OR 2.63, 95% CI 1.66–4.17), Quintile 4 (OR 2.17, 95% CI 1.39–3.38) and Quintile five (OR 2.90, 95% CI 1.84–4.58). No significant moderation effect was found between any pair of socio-demographic predictor variables.

**Table 3:** Logistic regressions predicting Health Check participation

*Variable*	*Odds ratio (95% confidence interval)*
Univariate logistic regressions	
Age	
Years	1.05 (1.04–1.07)**
Gender	
Male	1.00
Female	1.50 (1.16–1.95)*
Ethnicity^a^	
White	1.00
Other	0.59 (0.21–1.57)
IMD^b^	
Quintile 1 (least deprived)	1.00
Quintile 2	1.61 (1.03–2.51)*
Quintile 3	2.63 (1.66–4.17)**
Quintile 4	2.17 (1.39–3.38)**
Quintile 5 (most deprived)	2.90 (1.84–4.58)**
Multivariate logistic regression^c^	
Age	
Years	1.05 (1.04–1.07)**
Gender	
Male	1.00
Female	1.29 (0.95–1.76)
Ethnicity^a^	
White	1.00
Other	0.85 (0.29–2.52)
IMD^b^	
Quintile 1 (least deprived)	1.00
Quintile 2	0.56 (0.31–1.02)
Quintile 3	0.79 (0.41–1.52)
Quintile 4	0.37 (0.18–0.76)*
Quintile 5 (most deprived)	0.42 (0.20–0.88)*

For regression analyses, Health Check participation is coded as 1; Health Check non-participation is coded as 0.

^a^White Ethnicity (White British, White Irish, Other White Background); Other Ethnicity includes Mixed Ethnicity [Mixed White and Black Caribbean, Mixed White and Asian, Other Mixed Background, Asian Ethnicity (Asian or Asian British Indian, Asian or Asian British Pakistani, Asian or Asian British Bangladeshi, Other Asian Background), Black Ethnicity (Black or Black British Caribbean, Black or Black British African, Other Black Background), Chinese and Other].

^b^Index of Multiple Deprivation cut-off values for Quintile 1 = 6.12, Quintile 2 = 12.51, Quintile 3 = 16.23, Quintile 4 = 22.41.

^c^Adjusted for GP surgery.

*P < 0.05.

**P < 0.01 for Wald Statistic.

Multivariate logistic regression analyses, controlling for GP surgery, reduced to non-significant the association between gender and Health Check participation, whereas older age remained a significant predictor in this model (OR 1.05, 95% CI 1.04–1.07). IMD also continued to predict Health Check participation, although the direction of effect was reversed. Compared with the lowest IMD quintile, patients residing in more deprived Quintile four (OR 0.37, 95% CI 0.18–0.76) and Quintile five (OR 0.42, 95% CI 0.20–0.88) were now found to have significantly lower odds of taking part in the Health Check. Further analysis revealed that IMD distributions differed across the four practices, with a higher number of patients residing in areas in greater deprivation in Practices 3 and 2, and no patients in Quintile 5 in Practices 3 and 4 (*χ*2 = 731.21, df = 3, *P* < 0.005). In a subsequent multivariate logistic regression model including interaction terms, no significant moderation effects were found.

### Physical activity trial participants versus trial non-participants

Table 4 displays results of univariate and multivariate logistic regression analyses predicting trial participation (*n* = 1380). In univariate analyses, older patients (OR 1.03 95% CI 1.01–1.04) and women (OR 1.49, 95% CI 1.10–2.03) showed significantly greater odds of trial participation, whereas no significant difference was found between patients of white and other ethnicities. Compared with those living in areas of comparatively lowest deprivation, the odds of trial participation did not differ significantly across the remaining quintiles, with the exception of Quintile 4. Patients residing in this second most deprived quintile had around 40% lower odds of trial participation than their counterparts in Quintile 1 (OR 0.60, 95% CI 0.37–0.99). No significant interaction terms were found for any paired combination of socio-demographic predictor variables.

**Table 4:** Logistic regressions predicting physical activity trial participation

*Variable*	*Odds ratio (95% confidence interval)*
Univariate logistic regressions	
Age	
Years	1.03 (1.01–1.04)*
Gender	
Male	1.00
Female	1.49 (1.10–2.03)**
Ethnicity^a^	
White	1.00
Other	1.48 (0.72–3.04)
IMD^b^	
Quintile 1 (least deprived)	1.00
Quintile 2	0.74 (0.47–1.15)
Quintile 3	0.83 (0.52–1.33)
Quintile 4	0.60 (0.37–0.99)*
Quintile 5 (most deprived)	0.72 (0.45–1.17)
Multivariate logistic regression^c^	
Age	
Years	1.04 (1.02–1.06)**
Gender	
Male	1.00
Female	1.41 (1.00–1.99)
Ethnicity^a^	
White	1.00
Other	1.36 (0.64–2.91)
IMD^b^	
Quintile 1 (least deprived)	1.00
Quintile 2	0.70 (0.42–1.17)
Quintile 3	0.74 (0.41–1.32)
Quintile 4	0.62 (0.32–1.20)
Quintile 5 (most deprived)	0.61 (0.32–1.16)

For regression analyses, trial participation is coded as 1; trial non-participation is coded as 0.

^a^White Ethnicity (White British, White Irish, Other White Background); Other Ethnicity includes Mixed Ethnicity [Mixed White and Black Caribbean, Mixed White and Asian, Other Mixed Background, Asian Ethnicity (Asian or Asian British Indian, Asian or Asian British Pakistani, Asian or Asian British Bangladeshi, Other Asian Background), Black Ethnicity (Black or Black British Caribbean, Black or Black British African, Other Black Background), Chinese and Other].

^b^Index of Multiple Deprivation cut-off values for Quintile 1 = 6.12, Quintile 2 = 12.51, Quintile 3 = 16.23, Quintile 4 = 22.41.

^c^Adjusted for GP surgery.

*P < 0.05.

**P < 0.01 for Wald Statistic.

In the subsequent multivariate model, following adjustment for GP surgery, only age remained a significant predictor of trial participation, with a 4% increase in odds seen with every 1-year increase in age (OR 1.04, 95% CI 1.02–1.06). Women also continued to show greater odds of participation than men when controlling for all other variables, although this effect was borderline non-significant (OR 1.41, 95% CI 1.00–1.99, *P* = 0.053). Neither ethnicity nor IMD predicted trial participation in this model. No significant interaction terms were observed.

## Discussion

### Main findings of this study

This study aimed to explore whether participation in the NHS Health Check and a nested physical activity trial can be considered equitable by exploring differences in uptake across selected socio-demographic factors. In multivariate analyses controlling for GP surgery, participation in the Health Check (either alone or in addition to the trial) was predicted by older age and lower area-level deprivation. Participation in the physical activity trial component (nested within the Health Check) was predicted by older age. Together, these findings suggest that younger patients and those living in areas of relatively high socioeconomic deprivation may be less willing to take part in primary care-based preventative interventions, while younger (and possibly male) patients appear further disinclined to participate in research informing the development of these interventions. GP surgery exerted a substantial effect on the strength and direction of associations between socio-demographic variables and participation, a finding which suggests that practice-level factors may play a greater role in determining equity in participation than individual patient characteristics.

### What is already known on this topic?

Our finding that older individuals were more likely to participate in primary care-based preventative health interventions is supported by a number of existing studies conducted across varied settings and populations,^21,22^ including research specific to the NHS Health Checks Programme.^23,24^ Conflicting findings from older research studies do however also exist.^17^ A number of factors may explain the age association observed here, including the possibility of greater perceived relevance of preventative interventions in older groups given their increased risk profile^21^ or age-relevant issues surrounding access to GP appointments (e.g. older people may be less likely to be in full-time employment). The finding of a higher likelihood of attending a Health Check with lower levels of deprivation is consistent with the existing literature and suggests that more efforts may need to be directed towards understanding how best to engage disadvantaged social groups in preventative health interventions^17,19,20,23^.

When controlling for GP surgery within analyses, we observed differences in both the magnitude and, in the case of IMD, direction of the association between socio-demographic characteristics and participation. This finding highlights the importance of considering GP-surgery-specific factors when exploring reasons why patients choose to take part or otherwise in primary care-based interventions. We note, for example, disparities in patient IMD distributions across the GP surgeries included in this study, and the fact that each practice organized and conducted recruitment into the Health Check and trial in different ways. To date, a large body of research has highlighted the importance of aspects such as GP surgery size,^23,24^ GP patient ethnic concordance,^24^ recruitment strategies^34^ (for example, telephone, verbal or opportunistic invitations)^35,36^ and patient beliefs surrounding access to appointments^37^ in predicting Health Check uptake. Moreover, the nature of the referral process (e.g. the referring health professional and reasons for referral)^27,38,39^ and perceived physician support^40^ have also been shown to influence uptake of physical activity promotion trials and interventions conducted in primary care. Each surgery in the present study employed different recruitment approaches, ranging from postal invitation letters to face-to-face ad-hoc requests, and Health Check availability and delivery is likely to have differed in quantity, quality and frequency across surgeries. Unfortunately, information on recruitment procedures was not collected in a manner amenable to statistical analysis here, although other research programmes are currently exploring the impact of different invitation procedures on Health Check uptake.^34^

### What this study adds

This study is one of a few available analyses exploring equity in uptake of the NHS Health Check across patient socio-demographic factors.^24,41^ Since its inception in 2008, the Health Check programme has been criticized for failing to attain the originally projected participation rates upon which initial estimates of cost and clinical effectiveness were based.^42^ Further exploration of how potential socio-demographic determinants of Health Check uptake may be modified by GP surgery-related factors is now required to ensure that the programme not only achieves its aim of reducing vascular disease incidence in the population but does so without increasing health inequities between population subgroups.^42^ This latter priority has been highlighted in a number of existing publications examining uptake and response to the programme, with no clear conclusions on equity so far drawn.^36,43^

The findings of the present study suggest that a focus on younger patients residing in areas of greater socioeconomic deprivation may be a good starting point. Further work in this area may be usefully informed by the Cochrane & Campbell Equity Method Groups ‘PROGRESS-Plus’ equity checklist. This checklist highlights the need to expand equity considerations beyond more traditionally studied social stratifiers such as age, gender and socioeconomic status to consider a far broader range of factors known to typify social disadvantage (e.g. Place of residence, Race, Occupation, Gender, Religion, Education, Social Capital, Socioeconomic status, plus age, disability and sexual orientation)^44^. Further work, including qualitative research, is currently underway to explore how these factors may lead to differential uptake within the context of the Health Check and in primary care preventative interventions more generally.

### Limitations of this study

One key limitation of this study is the fact that participating practices were located in one geographic region of the UK (East of England) and served a largely racially homogeneous population of patients residing in areas of relatively low deprivation (both Practices 3 and 4 were in areas classified as falling within the least deprived IMD decile in the country)^33^. This constraint was due to the geographic catchment area of the pilot trial from which data were obtained. We acknowledge that further work exploring the role of GP surgery-related factors in determining equitable uptake of the Health Check is now required in more socially diverse populations and in larger patient samples. That differences in participation by age, IMD and gender were identified within the present patient population implies that larger effects may be observable in populations showing greater socio-demographic variation. Larger sample sizes may also increase the likelihood of detecting potential interaction effects between socio-demographic variables. This would allow us to explore how different indices of social disadvantage may potentially augment the effects of each other on health outcomes.

A further limitation is the fact that other PROGRESS-Plus factors and the pathways through which these may influence recruitment were not explored in the present study. This shortcoming resulted from ethical constraints surrounding access to patient records and from the limitations of demographic data stored on practice databases. Furthermore, we experienced difficulties measuring and operationalizing aspects of Health Check delivery such as ad hoc recruitment procedures. Recruitment of non-participants into research is, by definition, a difficult task, and we acknowledge that one strength of this work is that we were able to obtain data on a relatively large sample of practice-registered non-participants. We had originally hoped to include data from a larger number of practices in our analyses, but we were prevented from doing so by a lack of consistent record keeping on the numbers of patients invited to participate in the Health Check and trial. Further research in this area may benefit from engaging practice staff responsible for recruitment earlier on in the process to ensure that suitable data can be gathered on the numbers and characteristics of invited patients and recruitment procedures used.

To conclude, younger patients in the target 40–74 age year range may be at greater risk of non-participation in the NHS Health Checks programme and a nested physical activity trial. Lower socioeconomic status appears to be an additional risk factor for non-participation in the Health Check, while men may be less likely than women to take part in the research trial component specifically. GP surgery-related factors are key determinants of uptake in this context and need to be studied in greater detail to isolate which aspects of programme delivery encourage participation across different socio-demographic groups and thereby ensure equity in uptake.

## Funding

This paper presents independent research funded by the National Institute for Health Research (NIHR) under its Programme Grants for Applied Research Programme (Grant Reference Number RP-PG-0608-10079). The views expressed are those of the authors and not necessarily those of the NHS, the NIHR or the Department of Health.
